# Entropy-Based Correlation Analysis for Privacy Risk Assessment in IoT Identity Ecosystem

**DOI:** 10.3390/e27070723

**Published:** 2025-07-03

**Authors:** Kai-Chih Chang, Suzanne Barber

**Affiliations:** Department of Electrical and Computer Engineering, The University of Texas at Austin, Austin, TX 78712, USA; sbarber@identity.utexas.edu

**Keywords:** identity, privacy, privacy policy, Internet of Things, privacy risks, entropy

## Abstract

As the Internet of Things (IoT) expands, robust tools for assessing privacy risk are increasingly critical. This research introduces a quantitative framework for evaluating IoT privacy risks, centered on two algorithmically derived scores: the Personalized Privacy Assistant (PPA) score and the PrivacyCheck score, both developed by the Center for Identity at The University of Texas. We analyze the correlation between these scores across multiple types of sensitive data—including email, social security numbers, and location—to understand their effectiveness in detecting privacy vulnerabilities. Our approach leverages Bayesian networks with cycle decomposition to capture complex dependencies among risk factors and applies entropy-based metrics to quantify informational uncertainty in privacy assessments. Experimental results highlight the strengths and limitations of each tool and demonstrate the value of combining data-driven risk scoring, information-theoretic analysis, and network modeling for privacy evaluation in IoT environments.

## 1. Introduction

Ensuring the privacy and security of personal data in the rapidly growing Internet of Things (IoT) ecosystem is a critical challenge. Privacy risk assessment tools are essential for identifying potential vulnerabilities and mitigating privacy breaches in such systems [1]. This research introduces an analysis of two key privacy risk assessment tools: the Personal Privacy Assistant (PPA) score and PrivacyCheck score, both developed at Center for Identity at University of Texas at Austin (UTCID). These tools are designed to evaluate the privacy risks associated with various types of sensitive data, helping individuals and organizations assess vulnerabilities within IoT systems.

The PPA score provides a personalized measure of privacy risk by evaluating the context in which personal data is shared, while the PrivacyCheck score assesses the overall effectiveness of a system in protecting personal information from potential threats. In this study, we explore the correlation between these two scores, focusing on how they assess privacy risks for high-impact sensitive data types [2,3] such as email addresses, social security numbers (SSNs), and location data—attributes that, if compromised, could lead to significant personal or financial harm.

UTCID PPA incorporates a Bayesian network (BN) framework [4], which models the complex relationships between various privacy risk factors within the IoT ecosystem. Bayesian networks are ideal for modeling probabilistic dependencies between identity attributes, where nodes represent personally identifiable information (PII) attributes, and edges represent the conditional probabilities of one attribute’s exposure given another’s breach. Examples of PII attributes include “What you Know” (e.g., name, address), “What you Have” (e.g., credit card information), “What you Are” (e.g., biometric data), and “What you Do” (e.g., browsing history) [5]. This network enables dynamic, probabilistic assessments of privacy risk exposure.

While Bayesian networks are a powerful tool for modeling complex interdependencies in privacy risk assessments, the presence of cyclic dependencies within the network structure complicates inference. Cycles can introduce computational inefficiencies, obscure causal relationships, and lead to errors in risk predictions [6]. To address these challenges, we propose a cycle decomposition method to decompose these cycles into acyclic subgraphs, improving both inference accuracy and computational efficiency. This method plays a critical role in enhancing the privacy risk assessment process by ensuring that only relevant paths of potential identity exposure are analyzed.

Additionally, we introduce formal entropy-based metrics to quantify the informational uncertainty of privacy risk scores. By measuring entropy, we assess the consistency and informativeness of both the PPA and PrivacyCheck scores across different data types [7]. This approach offers a rigorous, information-theoretic perspective on privacy risk evaluation, enabling us to identify areas where risk assessment is ambiguous and to highlight where each tool provides the greatest or least certainty in detecting privacy vulnerabilities.

The primary contributions of this paper are as follows:

- Cycle Decomposition for Bayesian Networks:We present a systematic cycle decomposition method that transforms cyclic Bayesian networks into acyclic subgraphs, enabling accurate and efficient privacy risk inference for identity theft scenarios in IoT ecosystems.
- Entropy-Based Quantification of Uncertainty: We apply Shannon entropy to quantify the informational uncertainty of privacy risk scores, offering a rigorous, interpretable metric to evaluate the reliability of privacy risk assessments across data types.
- Comparative Analysis of Privacy Scores: We conduct an empirical analysis comparing the PPA score and PrivacyCheck score, using correlation, regression, and feature importance methods to identify their respective strengths and limitations for different data types.
- Alignment with Standard Privacy Frameworks: We map our automated risk assessment approach to established frameworks such as CNIL and LINDDUN, demonstrating how our tools operationalize key risk analysis steps at scale.
- Validation on Real-World Data: We validate our methods on an open-source IoT app dataset, illustrating practical applications for risk detection and policy improvement in real-world settings.

The remainder of this paper is organized as follows. Section 2 discusses related work and existing solutions for privacy risk assessment and cycle handling in Bayesian networks. Section 3 introduces the proposed solution, detailing the cycle decomposition method and entropy-based analysis. Section 5 presents the experimental results validating the effectiveness of the proposed method. Section 6 concludes the paper.

## 2. Related Work

### 2.1. Identity Theft Simulation

Identity theft simulation is a vital research area for understanding its dynamics and developing prevention and mitigation strategies. Various studies employ diverse simulation techniques to address this issue.

Agent-based modeling is widely used to simulate identity theft dynamics within complex systems. For example, ref. [8] used agent-based models to study individual and entity behaviors in identity theft incidents, identifying contributing factors and evaluating intervention strategies.

Social network analysis focuses on how identity theft propagates through connections. Studies like [9] have analyzed the impact of network structures on the spread of identity theft and its dynamics.

Machine learning techniques are increasingly applied to simulate identity theft, analyze historical data, and predict trends. For instance, ref. [10] employed machine learning algorithms to simulate patterns, leveraging large datasets to train models and assess prevention measures.

Statistical analysis identifies key factors and trends in identity theft incidents. Research such as [11] has used statistical methods to assess the prevalence of identity theft and evaluate intervention measures.

In summary, identity theft simulation integrates agent-based modeling, social network analysis, machine learning, and statistical techniques to deepen understanding and improve prevention and mitigation strategies.

### 2.2. Cyclic Bayesian Network

Addressing cycles in Bayesian networks has been a key focus for improving inference efficiency and accuracy. Various methods have been developed, offering unique approaches to this challenge.

Belief propagation algorithms, such as the sum–product algorithm and its variants, are commonly used for inference in cyclic networks. These algorithms leverage message-passing properties to compute marginal probabilities efficiently, even with cycles [12].

Dynamic Bayesian networks (DBNs) extend traditional Bayesian networks to model temporal dependencies and feedback loops. DBNs enable the analysis of dynamic systems with cyclic behavior [13].

Approximate inference techniques, including variational methods and Monte Carlo sampling, offer another approach. These methods address cyclic structures by trading off some accuracy for computational efficiency [14].

A notable strategy is decomposing cyclic structures into acyclic subgraphs, which simplifies inference. For instance, a multimodule Bayesian network has been proposed for root cause analysis in complex chemical processes [15]. Similarly, a modified Bayesian network approach has been applied to handle cyclic loops in diagnosing faults in the chemical process industry [16].

These approaches collectively offer versatile solutions, catering to diverse application requirements and computational constraints.

### 2.3. Privacy Risk Assessment Frameworks

A variety of established frameworks guide privacy risk analysis in both academic research and industry practice. Here, we summarize the most influential approaches and contrast them with our automated, data-driven methodology.

LINDDUN [17] is a structured privacy threat modeling framework that helps system designers identify and mitigate privacy risks throughout software development. LINDDUN covers a broad spectrum of threat categories—including linkability, identifiability, non-repudiation, detectability, information disclosure, content unawareness, and policy/consent non-compliance—using systematic diagrams and threat elicitation steps. While LINDDUN is comprehensive for manual privacy analysis, its focus is on design-time threat modeling rather than automated, large-scale assessment.

The CNIL Privacy Risk Methodology [18] provides a step-by-step process for identifying privacy threats, estimating impact and likelihood, and synthesizing these factors into an overall risk rating. This research operationalizes similar concepts—probability and severity of identity attribute exposure—while enabling automated and scalable evaluation across hundreds of applications.

ISO/IEC 27557 [19] offers international standards for privacy risk management in information systems, defining risk assessment procedures, stakeholder roles, and documentation best practices. It emphasizes comprehensive documentation and stakeholder engagement, serving as a reference for organizational policy but less focused on rapid, automated assessments.

The NIST Privacy Framework [20] introduces a flexible, risk-based approach for improving privacy through core functions, but does not prescribe specific risk scoring or automation methods.

While these frameworks provide rigorous methodologies and policy guidance, our approach is unique in its automation, granularity, and focus on empirical, data-driven scoring. As shown in Table 1, our PPA and PrivacyCheck scores map core steps from these frameworks to quantitative, attribute-level risk metrics suitable for large-scale, comparative analysis.

Our system is complementary to these frameworks: it enables rapid, repeatable, and fine-grained risk assessment, while remaining conceptually aligned with the core steps and best practices from LINDDUN, CNIL, and ISO standards.

### 2.4. Summary

The related work surveyed above spans identity theft simulation, Bayesian network inference, and privacy risk assessment frameworks. Table 2 summarizes these categories, highlighting their core strengths and areas where further advances are needed.

While identity theft simulation leverages diverse analytical techniques—including agent-based modeling, social network analysis, machine learning, and statistical analysis—to provide insight into attack dynamics and prevention, these approaches are often context-specific and may not scale easily to complex, real-world systems.

Research on cyclic Bayesian networks addresses critical computational challenges, offering solutions such as belief propagation, dynamic Bayesian networks, approximate inference, and cycle decomposition. These methods improve inference tractability but may trade off interpretability or require extensive domain expertise.

Privacy risk assessment frameworks such as LINDDUN, CNIL, ISO/IEC 27557, and the NIST Privacy Framework provide rigorous methodologies and policy guidance. However, most rely on manual analysis, qualitative scoring, and are less suited for automated, large-scale, or attribute-level risk evaluation.

Our work complements and extends these approaches by enabling automated, scalable, and granular privacy risk assessment. By integrating data-driven techniques with established principles, our methodology addresses key gaps in current practice—especially in terms of efficiency, attribute-level analysis, and reproducibility.

## 3. UTCID PPA: Entropy-Enhanced Cycle Decomposition for Privacy Risk Assessment in Bayesian Networks

### 3.1. Threat Model

Our privacy risk assessment framework is grounded in a clearly defined threat model that captures plausible adversarial scenarios in the mobile app and IoT ecosystem.

#### 3.1.1. Adversary Profile

We assume a primary adversary whose goal is to compromise the privacy of end users by accessing, inferring, or misusing identity attributes collected, processed, or shared by apps. The adversary may take the form of:

- External attacker: Eavesdropping on unencrypted communications, exploiting insecure APIs, or leveraging data breaches to obtain sensitive attributes (e.g., SSN, email, location).
- Malicious insider: An app developer or employee with legitimate access who misuses privileged information for unauthorized purposes.
- Third-party service: Entities with whom data is shared, either intentionally (analytics, advertising) or inadvertently (poor access controls).

#### 3.1.2. Capabilities

We assume the adversary can:

- Access data transmitted or stored by the app, subject to permissions and technical controls;
- Analyze app privacy policies and user permissions to infer potential exposure;
- Aggregate data from multiple sources to enhance re-identification or profiling.

#### 3.1.3. Limitations

The adversary is assumed not to have physical access to the user’s device or backend infrastructure beyond what is available via public APIs or their own app installations. Advanced persistent threats or state-level actors are out of the scope for this work.

#### 3.1.4. Relation to Risk Scores

Our risk scores (PPA and PrivacyCheck) quantify the likelihood and potential impact of privacy exposure under this threat model. High scores indicate greater risk of attribute exposure or insufficient policy controls, thereby highlighting vulnerabilities to adversaries with the described capabilities. The entropy metric, in particular, reflects the uncertainty faced by the adversary in reconstructing a complete identity profile from partial information.

### 3.2. Entropy as a Measure of Informational Uncertainty in Privacy Risk Assessment

In this research, we formally define the informational uncertainty associated with privacy risk using Shannon entropy [21], a fundamental concept from information theory. Given a discrete random variable *X* with possible outcomes $x_{1},x_{2},\ldots ,x_{n}$ and associated probabilities $p_{i}$, the entropy H(X) is defined as:

(1) $$H\left(X\right)=-\sum_{i=1}^{n}p_{i}\log_{2}p_{i}$$

In the context of our privacy risk assessment, *X* may represent the set of possible outcomes for an identity attribute’s exposure, the possible answers to a privacy policy question, or the distribution of predicted privacy risk scores for an IoT device. For example, if we model the exposure of an attribute (e.g., SSN) across all apps, the probability distribution $P(x_{i})$ could reflect the likelihood that each app exposes this attribute.

Example Calculation:

Suppose for the attribute “Location,” 40% of apps do not collect it, 30% collect with consent, and 30% collect without consent. Then, the entropy is:

(2) $$H\left(X\right)=-\left[0.4\log_{2}0.4+0.3\log_{2}0.3+0.3\log_{2}0.3\right]\approx 1.57$$

A higher entropy indicates greater uncertainty in how the attribute is handled across the ecosystem, suggesting less predictability for both users and attackers.

In our experiments, we apply this formalism to (1) quantify the uncertainty in risk exposure estimates for each attribute and (2) measure the model’s confidence in privacy policy evaluations. High entropy values may highlight attributes or policies with inconsistent practices or ambiguous risk, guiding future privacy improvements.

In summary, we use entropy not only as an abstract concept but as a quantifiable, application-specific metric, with each term directly derived from the observed probability distributions in our IoT privacy risk datasets.

### 3.3. UTCID ITAP

As described in Section 1, this research utilizes two key privacy risk assessment tools: the PPA score and the PrivacyCheck score.

While both tools measure privacy risk, the PPA score is more granular and personalized, considering the specific context of data sharing, while PrivacyCheck score provides a high-level evaluation of the system’s overall privacy measures. In this research, we compare and analyze these two tools by performing a correlation analysis to assess how they interact when applied to different data types. Next, we introduce the essential technologies leveraged by UTCID PPA.

The Identity Threat Assessment and Prediction (ITAP) project [11] is designed to analyze and predict identity theft patterns by building a structured repository of information extracted from identity theft cases. Developed by the UTCID, ITAP employs advanced text mining techniques and natural language processing (NLP) to extract structure data (also called ITAP dataset in this research) from publicly available news stories using Rich Site Summary (RSS) feeds, search engines, and reports like those from the Identity Theft Resource Center [22]. Its ultimate goal is to mitigate identity theft and fraud by understanding the processes used by criminals, the vulnerabilities they exploit, and the potential losses incurred.

ITAP focuses on three core objectives:

- Understanding Identity Theft Processes: Analyze methods used by identity thieves and fraudsters to identify patterns and vulnerabilities;
- Risk Assessment: Quantify the exposure risks of different types of identity attributes;
- Loss Estimation: Determine the monetary losses associated with identity attribute exposure, enabling the identification of high-risk attributes.

#### 3.3.1. Defining Identity Attributes

To effectively model identity theft, UTCID ITAP requires a clear and consistent representation of identity attributes that are frequently exploited by criminals. A preexisting list of identity attributes was manually curated, identifying commonly targeted data such as social security numbers, credit card details, email addresses, and phone numbers. This predefined list was created by:

- Reviewing identity theft literature and reports;
- Identifying identity attributes most frequently mentioned in news articles and case studies.

During the analysis, UTCID ITAP matches the identity attribute list against the textual content of news stories. Each matched attribute is recorded in the structured identity theft record, allowing for further analysis of its role in the theft process.

#### 3.3.2. Risk of Exposure for Identity Attributes

The risk of exposure for each identity attribute is calculated based on its frequency of occurrence in the analyzed news articles from RSS feeds, search engines, and the Identity Theft Resource Center [22]. This research hypothesizes that higher frequency indicates a greater likelihood of exposure during identity theft. The probability value of risk of exposure for each attribute is derived as follows: (1) The total frequency of an attribute across all analyzed articles is used to estimate its exposure probability. (2) This frequency is normalized to create a comparative risk score among all attributes. This approach highlights high-risk identity attributes, enabling targeted strategies to mitigate their exposure. Entropy-based analysis is applied here to measure the uncertainty in the risk scores by quantifying how much information the exposure frequency provides. Entropy provides a deeper understanding of how reliable the risk exposure estimates are and how much variability exists across different identity attributes [23,24].

#### 3.3.3. Monetary Loss Assignment

To quantify the financial impact of identity theft, UTCID ITAP extracts monetary loss information from news articles using named entity recognition (NER). Losses are represented in a unified format (e.g., converting “1 million dollars” to “$1,000,000”) and aggregated for each article. UTCID ITAP further refines loss estimation by assigning weights to identity attributes based on their frequency of occurrence in the article. The weighted formula for loss calculation is as follows:

(3) $$L_{i}=L_{\mathrm{tot}}\times \frac{f_{i}}{\sum_{j=1}^{n}f_{j}}$$

where:

- $L_{i}=$ Loss attributed to a specific identity attribute *i*;
- $L_{\mathrm{tot}}=$ Total loss mentioned in the article;
- $f_{i}=$ Frequency of the identity attribute *i* in the article;
- $\sum_{j=1}^{n}f_{j}=$ Sum of frequencies of all identity attributes in the article and *n* is the number of distinct identity attributes mentioned in the article.

The loss is also called the “Liability Value” in this research. This formula ensures that attributes mentioned more frequently are assigned higher proportions of the total loss, reflecting their critical role in the theft.

#### 3.3.4. Dependency Analysis

If an article states that a fraudster obtained a victim’s social security number (SSN) by first acquiring their name, a directed dependency is created from the Name to the SSN. This dependency indicates that the breach of one attribute facilitates the compromise of another. To quantify the strength of these dependencies, UTCID ITAP calculates the weight of each dependency as the conditional probability of one attribute being breached given the breach of another. This conditional probability is estimated based on co-occurrence frequencies in the news articles. Let $N_{ij}$ denote the number of articles in which both attributes *i* and *j* are breached (with *i* preceding *j*) and $N_{i}$ denote the number of articles in which attribute *i* is breached. Then:

(4) $$w_{ij}=\frac{N_{ij}}{N_{i}}$$

where:

- $w_{ij}$ is the estimated conditional probability that attribute *j* is breached, given that *i* is breached.

Through these methodologies, UTCID ITAP not only introduces a structured approach to understanding identity theft but also provides actionable metrics for assessing risks and losses and populates the ITAP dataset [25]. By leveraging these insights, businesses, consumers, and policymakers can make informed decisions to safeguard sensitive information and reduce vulnerabilities.

UTCID ITAP currently models over 7000 news stories reporting specific identity theft and fraud cases and populates a dataset containing more than 600 identity attribute data points. UTCID ITAP readily provides the initial risk of exposure, the intrinsic loss value for identity attribute, and the dependencies between identity attributes. To visualize these relationships and perform probabilistic analysis, the UTCID IoT Identity Ecosystem is essential.

### 3.4. UTCID IoT Identity Ecosystem

The UTCID IoT Identity Ecosystem is a robust, graph-based framework designed to understand, analyze, and mitigate the risks associated with identity attributes. Its interactive visualizations provide insights into the impact of specific breach events. This system addresses the growing challenge of identity theft, a widespread issue affecting millions globally, by mapping and analyzing the relationships between various identity attributes in both physical and digital realms. Identity theft often stems from the misuse of interconnected identity attributes, where the compromise of one piece of information can lead to cascading breaches. Understanding these relationships is essential to predict risks, assess liabilities, and devise preventive measures effectively.

The UTCID IoT Identity Ecosystem employs a Bayesian Network due to its ability to model complex, probabilistic relationships between identity attributes. Bayesian Networks are particularly suitable for this purpose because:

- Causal Dependencies: They effectively capture the direct and indirect dependencies between identity attributes;
- Probabilistic Reasoning: They allow for inference under uncertainty, which is critical in predicting the risk of exposure and estimating potential liabilities;
- Scalability: The framework can handle a large number of interconnected variables, making it ideal for modeling the intricate nature of identity ecosystems.

In order to address the issue of cycles in Bayesian networks while preserving the characteristics of the UTCID IoT Identity Ecosystem, which simulates identity fraudsters’ behavior, this research chose to decompose cycles into several possible paths (subgraphs), and for each possible path, a simulation was conducted to perform Bayesian inference and analysis. Paths are represented by a succession of connections in the network when the exposure of one identity attribute (A) leads to the exposure of identity attribute (B) with some probability, which leads to the exposure of identity attribute (C) with some probability, and so on.

The following is an outline of the steps involved in the proposed method:

1. Given a set of evidence (identity attributes to be exposed), detect all cycles along the paths;
2. Attempt to remove edges contained within those cycles and confirm the validity of the paths after removal;
3. Record all valid subgraphs and conduct a simulation for each subgraph;
4. Finally, integrate the results of all simulations.

Decomposing cycles in Bayesian networks into several subgraphs helps alleviate the issues caused by cycles because it eliminates the presence of cycles within these subgraphs. Consequently, the original Bayesian inference method can be applied to each subgraph individually, as the absence of cycles ensures the validity of the inference process. Entropy-based methods are integrated into this process to evaluate how much uncertainty is associated with the decomposition. By computing the entropy of the paths and subgraphs, we can determine the amount of information each subgraph contributes to the overall privacy risk assessment and identify the most reliable paths for further analysis. More details will be further elaborated in the following sections.

### 3.5. Cycle Decomposition Algorithm Description

The proposed cycle decomposition algorithm aims to break down cycles within a Bayesian network into smaller, acyclic subgraphs. A description of the steps involved in this algorithm is described below.

#### 3.5.1. Cycle Detection

Given a Bayesian network and a set of evidence, the algorithm starts by detecting all cycles present within the network along the specified paths. This step involves traversing the network and identifying closed loops of dependencies among variables.

#### 3.5.2. Edge Removal Attempt

Once cycles are detected, the algorithm attempts to remove edges that contribute to the formation of these cycles. Removing edges breaks the circular dependencies and transforms the cyclic graph into an acyclic one. The algorithm systematically evaluates each cycle and removes edges within them, one at a time, to test the validity of the resulting path.

#### 3.5.3. Path Validity Check

After removing edges, the algorithm verifies whether the resulting paths remain valid for Bayesian inference. This check ensures that the removal of edges does not compromise the integrity of the network structure or the validity of probabilistic reasoning. Path validity may be determined by confirming that essential dependencies are preserved and that no new conflicts or inconsistencies arise.

#### 3.5.4. Subgraph Construction

**Figure 1:**
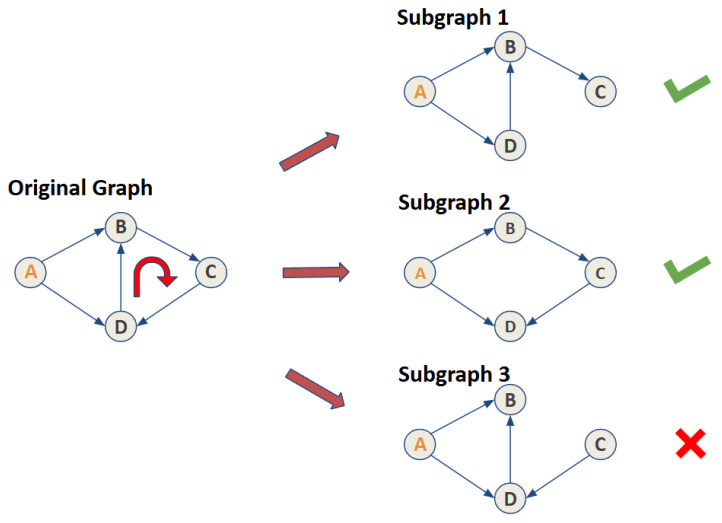
An example shows how a simple cycle can be decomposed into several subgraphs. This case also shows the validity of each subgraph with check marks.

When a path remains valid after edge removal, the algorithm records the resulting subgraph, representing a portion of the original Bayesian network with the cycle decomposed into smaller, acyclic components. Figure 1 illustrates this process using an example. Initially, the network comprises nodes A, B, C, and D, representing identity attributes in the UTCID IoT Identity Ecosystem, with edges denoting relationships. A cycle exists between nodes B, C, and D. Removing one edge decomposes the cycle into three potential subgraphs.

In each subgraph, edge removal alters node connectivity. The algorithm verifies validity by ensuring all nodes originally reachable from the evidence node (e.g., node A) remain accessible. For instance, in subgraphs 1 and 2, nodes B, C, and D remain reachable from node A, making these subgraphs valid. However, in subgraph 3, node C becomes unreachable due to the removal of the edge between B and C, rendering it invalid.

This process extends to cases involving multiple edge removals, generating additional subgraphs. Each undergoes a validity check to ensure all nodes originally reachable from the evidence node remain accessible. For example, removing both edges C–D and D–B yields a valid subgraph, since nodes B, C, and D remain reachable from A.

Variations in evidence nodes can influence subgraph validity. For instance, if both A and C are evidence nodes, subgraph 3 becomes valid as connectivity from the evidence nodes is preserved.

In summary, the process iteratively removes edges, evaluates subgraphs, and assesses their validity to decompose cycles in Bayesian networks.

#### 3.5.5. Simulation with Bayesian Inference on Subgraphs

Bayesian inference is a statistical framework for updating beliefs about unknown quantities based on observed data. In this research, it estimates the likelihood of identity attribute exposure and assesses the monetary loss associated with such exposure. The entropy of the posterior probability distributions resulting from Bayesian inference is used to quantify the uncertainty in the estimated privacy risks, providing a measure of the model’s confidence in its predictions.

Each identity attribute *i* has two key parameters: the prior probability P(i), representing the initial likelihood of exposure, and the liability value L(i), quantifying the maximum potential monetary loss if *i* is exposed. These parameters are updated through Bayesian inference using evidence of exposure events, refining beliefs about exposure likelihood and liability values [26].

##### Accessibility

In the context of Bayesian inference, the dynamic properties of an identity attribute are evaluated by examining its probability of exposure under different conditions. Let A(i) denote the set of ancestor attributes of identity attribute *i* in the Bayesian network.

For each ancestor $i_{k}\in A\left(i\right)$, we consider the conditional probability that *i* is exposed given that $i_{k}$ has been exposed, denoted $P(i|i_{k})$. The increase in exposure probability for *i* due to the exposure of $i_{k}$ is thus $P\left(i|i_{k}\right)-P\left(i\right)$, where P(i) is the marginal (prior) probability of *i* being exposed.

The Accessibility of attribute *i* is then defined as the sum of these increases over all its ancestors:

(5) $$\alpha \left(i\right)=\sum_{i_{k}\in A\left(i\right)}\left[P\left(i|i_{k}\right)-P\left(i\right)\right]$$

where α(i) reflects how easily *i* can be accessed indirectly through the exposure of its ancestors. A higher value of α(i) indicates greater overall risk of exposure due to indirect paths, while a lower value suggests that *i* is less accessible through such dependencies. The size and structure of A(i) directly influence the value of α(i), as a larger set of ancestors implies more possible entry points for exposure.

To further assess the reliability of these predictions, one can also evaluate the entropy of the set ${P(i|i_{k})}_{i_{k}\in A\left(i\right)}$, with higher entropy indicating more uncertainty in the conditional exposure estimates.

##### Post Effect

The second dynamic property, termed the Post Effect, describes how the exposure of a given identity attribute influences the risk profile of its descendants in a network, similar to risk propagation models widely used in network science and security risk assessment. Specifically, after a breach of attribute *i*, the increased exposure probability and associated loss among all downstream (descendant) nodes quantifies the cascading effect. This approach is consistent with prior work in risk analysis, where propagation of risk through network structures is modeled to evaluate both the direct and indirect consequences of initial events [27,28].

Let D(i) denote the set of descendants of *i* in the BN. For each descendant $i_{k}\in D\left(i\right)$, consider the conditional probability $P(i_{k}|i)$, representing the probability that $i_{k}$ is exposed given that *i* has already been exposed. The increase in the exposure probability for $i_{k}$ due to the exposure of *i* is then $P\left(i_{k}|i\right)-P\left(i_{k}\right)$, where $P(i_{k})$ is the marginal (prior) probability that $i_{k}$ is exposed.

Here, the liability value L(i) denotes the estimated monetary loss (defined at Equation (3)) associated with the exposure of identity attribute *i*. Assuming each identity attribute $i_{k}$ is associated with a liability value $L(i_{k})$, the increase in expected loss for $i_{k}$ resulting from the exposure of *i* can be written as $L\left(i_{k}\right)\cdot \left[P\left(i_{k}|i\right)-P\left(i_{k}\right)\right]$.

Thus, the total Post Effect for attribute *i* is defined as:

(6) $$\beta \left(i\right)=\sum_{i_{k}\in D\left(i\right)}L\left(i_{k}\right)\cdot \left[P\left(i_{k}|i\right)-P\left(i_{k}\right)\right]$$

where β(i) reflects the cumulative increase in expected monetary loss among all descendants of *i* as a result of its exposure. A higher value of β(i) indicates that the exposure of *i* can cause significant downstream risk, highlighting the attribute’s role as a potential risk propagator within the UTCID IoT Identity Ecosystem.

##### Privacy Risk

Privacy risk is defined as the potential monetary loss an individual may incur if specific identity attributes are compromised. The privacy risk score for each attribute quantifies this expected loss, with higher scores indicating greater potential for financial harm [29].

In this framework, each identity attribute *i* is assigned a liability value L(i), representing the estimated monetary loss associated with its exposure. The probability P(i) denotes the marginal (prior) probability that *i* will be exposed.

To capture both static and dynamic risk factors, two network-derived properties are incorporated:

- Accessibility (α(i)): The increase in exposure probability due to the compromise of *i*’s ancestors;
- Post Effect (β(i)): The cumulative increase in expected loss among *i*’s descendants if *i* is breached.

The expected loss for identity attribute *i* is given by:

(7) $$E\left(i\right)=P\left(i\right)\times \left[L(i)+L(i)\cdot \alpha (i)+\beta (i)\right]$$

where:

- P(i): Prior probability that *i* is exposed;
- L(i): Liability value (property damage) for *i*;
- α(i): Accessibility factor for *i*;
- β(i): Post Effect of *i*.

Here, L(i)·α(i) represents the additional expected loss attributable to increased exposure from *i*’s ancestors, while β(i) adds the downstream losses incurred by *i*’s descendants.

Because the expected loss E(i) can vary widely (from 0 up to $10^{7}$ in the UTCID ITAP dataset), a natural logarithm transformation is applied to compress its range for analysis. To enable comparison across attributes, the normalized privacy risk score is defined as:

(8) $$S\left(i\right)=\frac{\ln\left(E(i)\right)}{\ln\left(E_{\max}\right)}$$

where $E_{\max}$ is the maximum expected loss observed in the dataset. This normalization ensures that S(i)∈[0,1] for all attributes.

#### 3.5.6. Integration of Results

After applying Bayesian inference to each subgraph, various statistics are obtained for each node within each subgraph. These statistics include the prior change, accessibility, Post Effect, and privacy risk associated with an identity attribute in a subgraph. Additionally, the total privacy risk of a subgraph can be calculated to identify the most and least risky subgraphs. Furthermore, the sum of the weights of the edges in a subgraph can be determined to identify the most and least probable subgraphs.

Moreover, the average prior change, accessibility, Post Effect, and privacy risk for an identity attribute can be computed across all subgraphs. This average is interpreted as the result of the cyclic graph resulting from those subgraphs.

In summary, Bayesian inference yields various statistics for each subgraph, enabling the assessment of privacy risks and probabilities associated with different subgraphs. Additionally, entropy is used throughout the process to evaluate the uncertainty in the privacy risk predictions, ensuring that the cycle decomposition algorithm preserves the Bayesian network’s essential characteristics while enhancing probabilistic reasoning.

### 3.6. UTCID PrivacyCheck^™^: Privacy Policy Evaluation and Entropy-Based Risk Assessment

PrivacyCheck^™^ is an advanced machine learning-based privacy-enhancing tool developed by UTCID, designed to evaluate and score the privacy policies of IoT resources and other online services. This tool plays a critical role in helping users understand the data-sharing practices outlined in the privacy policies of IoT devices attempting to connect to their systems. By utilizing deep learning models trained on a wide range of privacy-related metrics, PrivacyCheck^™^ provides an intuitive assessment of a resource’s privacy policy, allowing users to make more informed decisions about data sharing [30,31].

PrivacyCheck^™^ evaluates privacy policies against a set of established privacy standards and regulations, such as the General Data Protection Regulation (GDPR) [30,32] and Fair Information Practice Principles (FIPPs) [33], as outlined by international and governmental bodies. The tool uses 20 carefully curated questions [34], as shown in Table 3, covering key areas like User Control, consent, data retention, third-party data sharing, and transparency. Each question is designed to assess the policy’s adherence to best practices in data protection and privacy management.

The scoring system is based on an easy-to-understand stoplight scheme, with scores ranging from green (indicating strong privacy practices) to red (indicating weak privacy practices). The results from PrivacyCheck^™^ offer a User Control score and a GDPR score, which provide a high-level overview of how well a privacy policy aligns with user-centric privacy protections and legal requirements. Additionally, the tool’s scoring methodology is powered by LightGBM (Light Gradient Boosting Machine), a machine learning technique that has been trained to predict the compliance of privacy policies based on the answers to these 20 questions.

In this research, we quantify the uncertainty inherent in PrivacyCheck’s privacy risk assessments using Shannon entropy [21]. Shannon entropy provides a formal measure of unpredictability associated with a discrete random variable or a probability distribution.

Let *X* be a discrete random variable representing the possible answer categories to a privacy policy question, and let $p=(p_{1},\ldots ,p_{n})$ be the corresponding probability distribution, where $p_{i}=P\left(X=x_{i}\right)$ and *n* is the number of possible answers. The Shannon entropy H(X) in Equation (1) can also be written as:

(9) $$H\left(X\right)=-\sum_{i=1}^{n}p_{i}\log p_{i}$$

where H(X) is the entropy (in nats if using the natural logarithm) and $p_{i}$ is the probability of the *i*-th answer category.

A higher value of H(X) indicates greater uncertainty in the model’s prediction for that question, while a lower value reflects more confidence or determinism. The entropy can equivalently be written as H(p) to emphasize it as a function of the probability distribution vector.

The overall uncertainty across all questions is quantified by summing the entropies for each question:

(10) $$H_{\mathrm{tot}}=\sum_{q=1}^{Q}H\left(X_{q}\right)$$

where *Q* is the total number of questions and $X_{q}$ is the random variable for the *q*-th question.

This quantitative measure provides insight into the stability and reliability of PrivacyCheck’s assessments. High entropy may indicate ambiguous or poorly defined privacy practices, whereas low entropy suggests the model can confidently evaluate a policy. By integrating entropy into our analysis (as described in Section 3.2), we identify areas needing further clarification or transparency, thus improving both the interpretability and effectiveness of privacy risk evaluation.

PrivacyCheck has been successfully applied in various privacy studies. For instance, it has been used to assess the impact of GDPR on the landscape of privacy policies [30], compare privacy policies across the public and private sectors [35], and analyze patterns in the use of privacy-enhancing technologies (PETs) [36]. By employing entropy in conjunction with traditional scoring methods, PrivacyCheck enables a deeper, more nuanced analysis of privacy policies, fostering better decision making for users concerned about their data privacy in IoT environments.

### 3.7. Summary of Notation

Table 4 summarizes all symbols and notations used in this section.

## 4. Evaluation

### 4.1. Comparison of Existing Approaches for Dealing with Cycles in Bayesian Networks

Addressing cycles in Bayesian networks is a challenging problem that has led to the development of various approaches, each with its strengths and weaknesses. The existing methods for handling cycles generally fall into the following categories: moralization with triangulation (used in the Junction Tree Algorithm), dynamic Bayesian networks (DBNs), cycle decomposition, and approximate inference techniques like loopy belief propagation.

Dynamic Bayesian Networks are an extension of Bayesian networks that model temporal processes by representing different time slices, with cyclic dependencies occurring over time [13].

Pros:

- Suitable for systems that evolve over time, making it ideal for temporal processes;
- Handles feedback and recursions by unrolling the network over multiple time steps.

Cons:

- Computationally expensive as the number of time slices increases, posing challenges for real-time decision making;
- May fail to capture necessary feedback loops concisely in less temporally structured environments like identity theft;
- Requires careful temporal modeling, adding complexity.

Loopy belief propagation is an approximate inference technique that works by iteratively passing messages through the network, even with cycles [12].

Pros:

- Faster than exact methods like the Junction Tree Algorithm and can work well for very large networks;
- Handles cyclic dependencies without converting the network to an acyclic form.

Cons:

- Results are approximate and may converge to incorrect beliefs;
- Struggles with maintaining causal interpretability, which is critical in identity theft scenarios.

Moralization with Triangulation (Junction Tree Algorithm) converts the directed, cyclic Bayesian network into an undirected one by moralizing the graph (adding undirected edges between parents of common children) and then triangulating it to remove cycles, forming a junction tree for exact inference [37].

Pros:

- Provides exact inference, ensuring high accuracy;
- Effectively manages large, complex systems by breaking them into manageable cliques.

Cons:

- Triangulation can exponentially increase clique sizes, raising computational costs;
- Obscures causal relationships and feedback loops, reducing interpretability for cyclic processes like identity theft.

Cycle Decomposition method breaks the Bayesian network into smaller, acyclic subgraphs, preserving the original structure and feedback loops [15].

Pros:

- Preserves feedback loops and causal structures critical for applications like identity theft simulation;
- Useful for applications where cycles reflect natural processes (e.g., feedback loops in systems or processes). In the IoT Identity Ecosystem example, simulating cyclic criminal behavior makes this method more realistic;
- Realistically simulates scenarios where cycles reflect natural processes;
- Flexible and customizable for domain-specific needs.

Cons:

- Decomposing cycles requires domain expertise and can be computationally complex in larger networks;
- Relationships and dependencies across the entire network may be harder to infer compared to structured methods like the junction tree;
- Lacks standardized, widely accepted formal algorithms.

#### Why Some Methods Are Not Ideal for Identity Theft Scenarios

Identity theft involves dynamic feedback loops between identity attributes, behaviors, and outcomes. For instance, a stolen piece of information can trigger cascading effects, creating a feedback loop of identity compromise. Traditional methods like moralization with triangulation or DBNs often obscure these feedback loops or impose computational burdens that hinder real-time inference.

Cycle decomposition is particularly suited for identity theft scenarios as it preserves the network’s causal structure without losing feedback loops, which are essential for understanding and simulating how identity theft propagates. This method:

- Preserves causal relationships: Retains the network’s original structure, crucial for tracking cause-and-effect dynamics;
- Enhances interpretability: Maintains visibility into decision paths and feedback processes, helping analysts understand interactions between identity attributes and risks;
- Improves efficiency: Focuses on cyclic parts of the network, offering a more computationally feasible solution than triangulation.

In summary, while the Junction Tree Algorithm excels in exact inference, its treatment of cycles and feedback loops makes it less ideal for identity theft scenarios. Cycle decomposition, with its ability to retain recursive structures and causal relationships, emerges as the optimal choice for simulating and mitigating identity theft risks.

### 4.2. Exploratory Comparison with Privacy Risk Score Algorithm and ImmuniWeb®

To provide additional context for our PPA scoring algorithm, we conducted an exploratory comparison against an established, industry-recognized application security assessment tool: ImmuniWeb® Mobile App Scanner [38]. ImmuniWeb leverages AI and machine learning for automated security testing, with a particular focus on application vulnerabilities and privacy exposures. For this analysis, we selected ImmuniWeb’s risk categories most relevant to privacy, such as the detection of potentially sensitive data exposure. Each app assessed by ImmuniWeb receives a risk classification—“High”, “Medium”, “Low”, or “Warning”—indicating the severity of identified privacy or security issues, with “High” reflecting the greatest concern.

It is important to note, however, that ImmuniWeb and the PPA model fundamentally different aspects of privacy risk. ImmuniWeb evaluates app-level technical security and privacy vulnerabilities via static and dynamic analysis, whereas the PPA quantifies user-level contextual privacy risk using a Bayesian network approach. As such, any observed consistency between PPA scores and ImmuniWeb risk classifications should be understood as a qualitative correlation rather than formal validation.

Our evaluation focused on a subset of widely used mobile applications, each exceeding five million downloads according to Google Play statistics. For these popular apps, we computed both the PPA score and recorded the corresponding ImmuniWeb risk classification. Table 5 presents the results, with apps ordered by their PPA risk score.

The comparison reveals that applications assigned higher PPA scores (indicating elevated privacy risk) often received more cautionary or higher-risk labels from ImmuniWeb. While this alignment is noteworthy, we emphasize that it does not constitute external validation of the PPA approach. Rather, it offers exploratory evidence that the PPA’s user-level risk modeling may be complementary to established technical app assessment tools, providing additional perspective on privacy risk in real-world app ecosystems.

### 4.3. Validation of PrivacyCheck

To assess the accuracy and utility of PrivacyCheck’s automated privacy policy summarization, we conducted a multiphase evaluation using both cross-validation and head-to-head comparison with established external tools and manual ground truth.

**Model Training and Cross-Validation.**

PrivacyCheck uses supervised machine learning models to assign risk levels (green, yellow, red) for ten key privacy factors in website privacy policies. These models were trained on a corpus of 400 privacy policies sampled from major public companies (NYSE, Nasdaq, AMEX) and labeled by a multiperson expert team. Each model’s predictive accuracy was estimated via five-fold cross-validation, yielding correct label assignment rates ranging from 40% to 73%, depending on the factor (see Table 6). This confirms that PrivacyCheck is able to automatically identify risk levels for most privacy factors with moderate to high accuracy, even on unseen policies.

**Testing on Unseen Policies.**

To further validate generalizability, PrivacyCheck was tested on 50 new privacy policies not used during training. Each policy was manually reviewed to create a ground-truth answer key for all ten factors. PrivacyCheck’s automated assessments matched the ground truth between 42% and 76% of the time, consistent with cross-validation performance and supporting its robustness.

**Comparison with Alternative Tools.**

PrivacyCheck’s coverage and accuracy were also benchmarked against leading privacy policy analysis tools in the literature, including P3P (Platform for Privacy Preferences), ToS;DR (crowdsourced ratings), Privee (machine learning), and Usable Privacy (NLP annotation). PrivacyCheck demonstrated broader applicability and greater automation than tools such as P3P and ToS;DR, which suffer from limited coverage (only 1 of 50 policies was available for P3P, and 14 for ToS;DR). In direct comparison on the test set, PrivacyCheck achieved an average accuracy of 60%, compared to 74% for Privee, with both tools exceeding the coverage and scalability of crowdsourced or format-dependent solutions (see Table 7).

**User Adoption and Feedback.**

PrivacyCheck has been adopted by over 400 independent Chrome users, with positive user ratings and feedback affirming its usability and real-world value in helping users rapidly understand privacy practices without reading full policies.

Collectively, these results demonstrate that PrivacyCheck offers an accurate, scalable, and practical solution for summarizing privacy policies, with validated performance comparable to, or exceeding, many established tools and approaches in the field.

### 4.4. Comparison with the CNIL Privacy Risk Methodology

The CNIL Privacy Risk Method [18] is a widely used framework that structures privacy risk assessment into four key steps: (1) identifying privacy threats, (2) assessing the severity of potential impacts, (3) estimating the likelihood of occurrence, and (4) combining these to determine the overall risk level.

Our proposed scoring systems, PPA and PrivacyCheck, align closely with these core elements while offering an automated, data-driven, and scalable approach to privacy risk evaluation. Table 8 summarizes how our tools map onto each stage of the CNIL methodology.

Both PPA and PrivacyCheck automate and quantify the main stages of the CNIL risk assessment process. The PPA score delivers a granular, attribute-level risk analysis, while PrivacyCheck evaluates the adequacy of an app’s policy controls. Although our methods focus on automation and large-scale analysis, rather than manual case-by-case assessment, they are conceptually aligned with CNIL’s framework. Limitations include the absence of certain organizational or contextual factors considered in full CNIL analyses; future work may incorporate these for even greater fidelity.

In addition to validating our scores against internal metrics, we compared PPA and PrivacyCheck with established tools and frameworks, as described above. These external validations demonstrate both the effectiveness and the distinct focus of our automated approach relative to prior work.

## 5. Experiments and Results

### 5.1. Experimental Setup

To investigate the relationship between privacy risk and compliance-based policy scores, we conducted an empirical analysis over 200 open-source Android applications. The experimental pipeline integrates identity attribute extraction, privacy risk assessment, and policy evaluation, structured as follows:

- Application Dataset: We used a dataset of 200 open-source Android apps, each annotated with metadata including app category, link to the apps, and permission usage. Apps were chosen using a stratified sampling approach to capture a representative cross-section of market-leading and emerging applications. The sample includes both well-established and lesser-known apps to evaluate the generalizability of our risk assessment models.
- Identity Attribute Inference: For each app, we analyzed the AndroidManifest.xml file to determine the permissions it requests. We mapped each permission to one or more identity attributes based on a predefined ontology. Additionally, we parsed each app’s privacy policy to identify explicitly declared identity attributes with Natural Language Processing (NLP). The union of permissions and declared attributes was treated as the complete set of identity attributes collected by the app.
- PPA Risk Score Calculation: Using the inferred identity attributes, we computed a PPA risk score for each app (cycle decomposition included). This score captures both the exposure likelihood and the consequence of breach for the set of collected attributes, based on the UTCID IoT Identity Ecosystem model.
- Privacy Policy Evaluation via PrivacyCheck: We applied the UTCID PrivacyCheck^™^ tool to each app’s privacy policy. PrivacyCheck uses machine learning to answer 20 predefined questions grounded in FIPPs and GDPR principles. This produces both an overall User Control score and GDPR compliance score, as well as scores for individual privacy provisions.

This setup enables a fine-grained comparison between user-facing privacy assurances and underlying technical privacy risks across a diverse set of mobile applications. In the following subsections, we first show the cycle decomposition result and then we show the correlation analysis for the PPA score and PrivacyCheck score.

### 5.2. Cycle Decomposition Result Analysis

**Figure 2:**
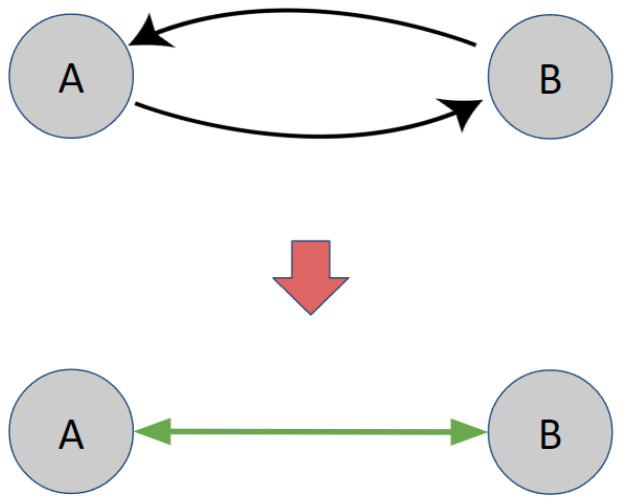
A diagram illustrating the simplification of a two-node cycle into a bidirectional edge. Node A and node b are nodes in UTCID IoT Identity Ecosystem. Arrows are the direction of the edge. Gray edges are directional edges. Green edge is bidirectional edge.

**Figure 3:**
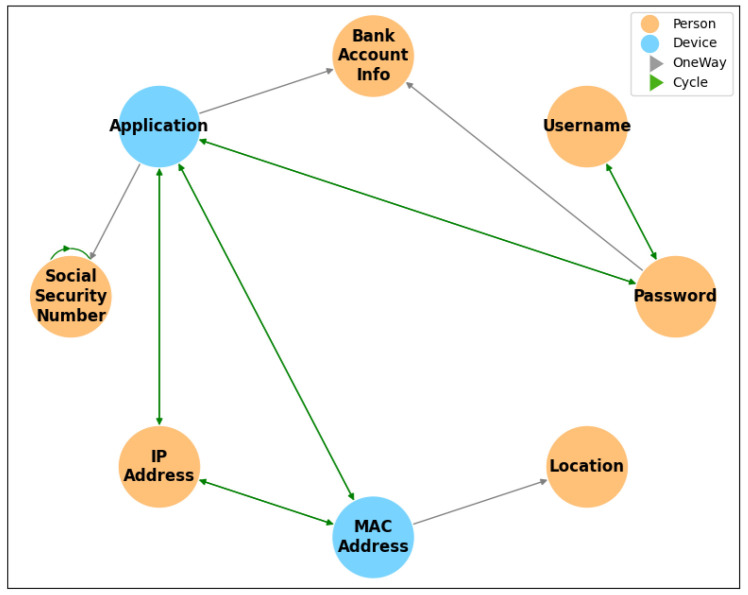
Graph representing a subset of the IoT Identity Ecosystem, comprising eight identity attributes. Blue nodes represent identity attributes for devices, while orange nodes represent identity attributes for people. The graph includes simplified bidirectional edges resulting from cycle reduction.

To simplify the evaluation of the proposed method, experiments were conducted on a small subset of the UTCID IoT Identity Ecosystem. Cycles formed by two nodes are represented as single bidirectional edges, depicted in green, while unidirectional edges are shown in gray ( Figure 2). The experimental graph ( Figure 3) includes six identity attributes for people (Password, Username, Bank Account Information, SSN, IP Address, Location) and two for devices (MAC Address, Application). Blue nodes represent device attributes, and orange nodes represent people’s attributes. The graph features six green edges, including cycles formed by the SSN node, which are decomposed using the proposed method.

**Figure 4:**
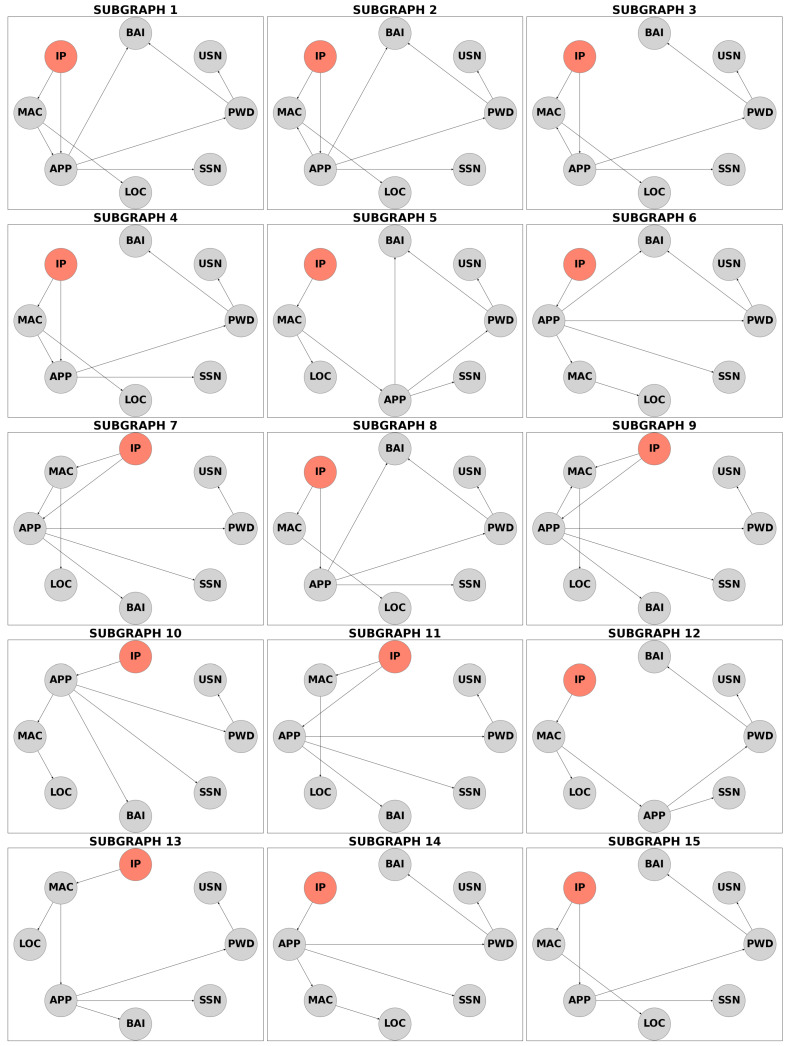
Decomposition results for the input network with the red node indicating the evidence (the node that has been breached/exposed). To improve readability with enlarged node labels, commonly used attribute names have been abbreviated (e.g., “IP Address” as “IP”, “Username” as “USN”, “MAC Address” as “MAC”, “Application” as “APP”, “Password” as “PWD”, “Location” as “LOC”, “Bank Account Information” as “BAI”, and “Social Security Number” as “SSN”).

The IP Address node was used as evidence, representing a breach or exposure. The method first detects cycles within the graph, including self-cycles, two-node cycles, and larger cycles. Edges are removed iteratively, and after each removal, the algorithm verifies graph validity. Valid subgraphs are those where the evidence node can still reach all other nodes. This process identified 15 valid subgraphs, indicated in Figure 4. For simplification and readability, all nodes are depicted in gray, with only the evidence node highlighted in red to indicate the starting point. All edges in Figure 4 are directed because all cycles have been removed. Subsequently, simulations can be performed separately for each of these cycle-free subgraphs.

Bayesian inference was performed on each subgraph, with results summarized in Table 9. Subgraphs S1 to S15 show metrics such as “Prior Change,” “Edge Weight,” “Accessibility,” “Post Effect,” and “Privacy Risk,” along with their averages (AVG). Row values are normalized, with the maximum value in each row displayed as 1.00.

Key findings include:

- Prior Change: Subgraph 8 shows the highest impact on priors following an IP Address breach, while subgraph 14 has the least;
- Edge Weight: Subgraph 2 is the most probable path for fraudsters, while subgraph 13 is the least likely;
- Accessibility: Subgraph 12 indicates the highest likelihood of access by other nodes, whereas subgraph 1 has the lowest;
- Post Effect: Subgraph 15 reflects the highest monetary loss post breach, while subgraph 11 shows the lowest;
- Privacy Risk: Subgraph 10 poses the greatest cumulative risk, while subgraph 1 represents the least.

This streamlined analysis highlights the nuanced impacts of subgraph configurations in evaluating privacy risk.

### 5.3. Correlation and Regression Analysis

Pearson correlation analysis revealed a strong positive correlation between GDPR scores and PPA risk scores (r ~ 0.55, *p* < 0.001), counterintuitively suggesting that higher compliance claims align with greater privacy risks. Regression analysis (Table 10) confirmed that GDPR scores were statistically significant predictors of PPA risk, whereas Control scores showed weaker and often non-significant associations after filtering out outliers (Control Overall Score = −50).

**Figure 5:**
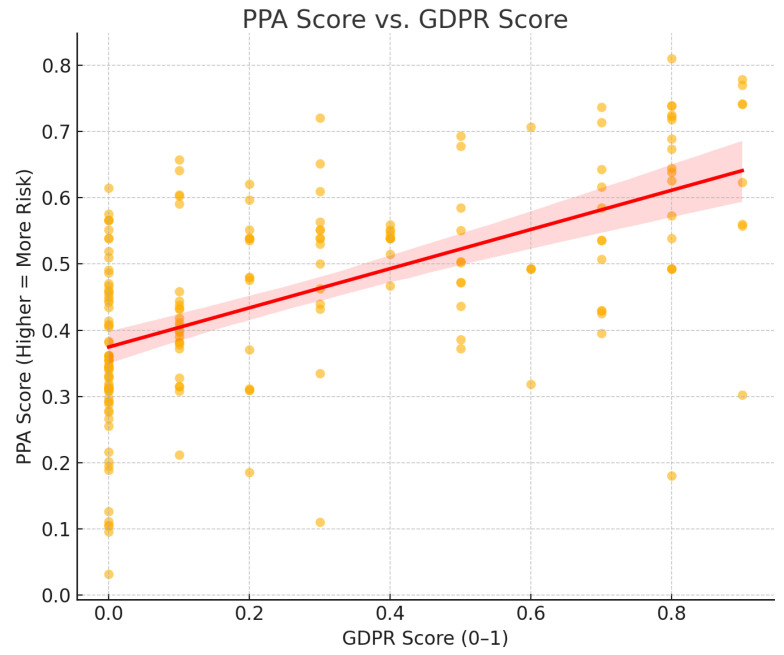
Scatter plot for GDPR score and PPA score. The red shaded area around the line indicates the region where the true regression line is expected to fall with a 95% confidence.

Figure 5 visualizes this relationship as a scatter plot. A clear upward trend confirms that apps with higher GDPR compliance scores can still pose significant real-world privacy risks.

The observed positive correlation between GDPR compliance scores and privacy risk may be explained by behavioral factors in user decision making. In real-world scenarios, users often rely on surface-level privacy indicators when evaluating whether to share personal information with an IoT device or app.

Consider the following plausible sequence of events:

1. A user is alerted by the PPA that connecting to a particular IoT device or application carries a high privacy risk due to the types of identity attributes it collects.
2. The user then reviews the associated PrivacyCheck scores for the app or device. If the GDPR compliance score is high, the user may infer that the company has a strong privacy policy and adheres to regulatory standards.
3. This perception can enhance the user’s trust in the company’s data handling practices, regardless of the underlying technical exposure. As a result, the user may proceed to share personal information despite the PPA’s high-risk assessment.

This behavioral dynamic helps explain why high GDPR scores are often associated with high privacy risk scores: users may be more inclined to trust and engage with apps that appear compliant, even when those apps collect more sensitive or extensive personal data. This highlights a crucial gap between perceived policy quality and actual privacy exposure.

These results reveal a potential disconnect between formal privacy compliance and actual risk exposure in IoT applications. The strong, statistically significant relationship between GDPR scores and PPA risk scores suggests that organizations may focus on meeting compliance checklists without necessarily reducing the underlying privacy risks for users. In practice, this means that users—and even regulators—may be lulled into a false sense of security based solely on policy documentation, overlooking technical and behavioral realities. Our findings underscore the importance of supplementing policy-based assessments with empirical, data-driven risk metrics when evaluating IoT privacy threats.

### 5.4. Risk Band Distribution and PrivacyCheck Scores

To analyze how privacy compliance correlates with empirical risk, we categorized applications into four risk bands based on their PPA risk scores: Low (0–0.25), Moderate (0.26–0.5), High (0.51–0.75), and Critical (0.76–1.0).

**Figure 6:**
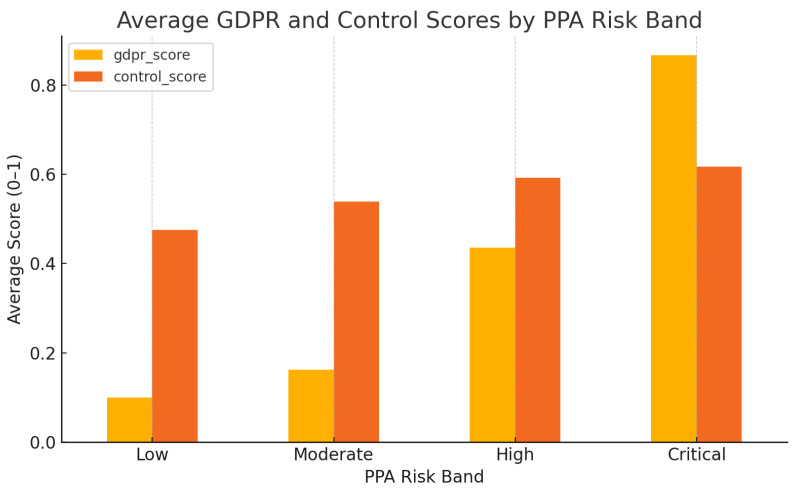
Average GDPR and User Control scores across PPA risk bands.

Figure 6 illustrates the average GDPR and User Control scores within each band. Notably, applications within the Critical risk band often maintained high GDPR compliance scores, suggesting that stated policy adherence does not necessarily guarantee lower empirical privacy risks.

**Figure 7:**
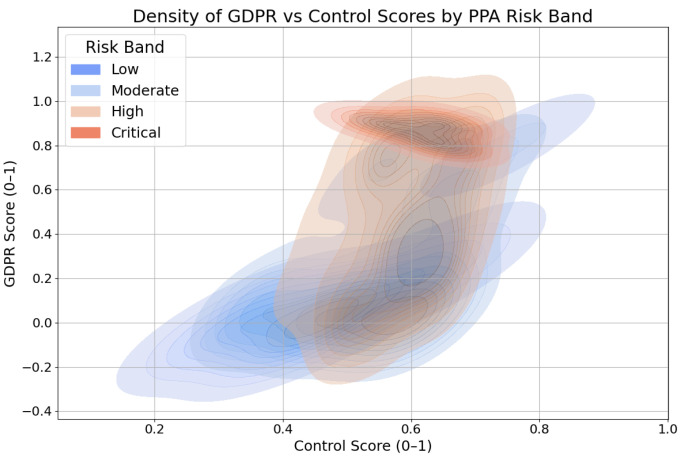
Density of GDPR vs. Control scores by PPA risk band.

To further illustrate the distribution of compliance characteristics by risk level, Figure 7 presents a density plot of GDPR versus Control scores colored by PPA risk band. This plot highlights that Critical-risk apps can be found across the full spectrum of GDPR and Control scores, indicating substantial overlap in policy representations regardless of actual risk.

The overlap of high-risk apps across all GDPR and Control score ranges highlights a critical weakness in current policy-centric evaluation approaches. Even apps that appear highly compliant on paper may, in reality, expose users to substantial privacy threats. This finding reinforces the need for combined policy and technical analysis when performing risk assessments, as relying on compliance indicators alone may not adequately protect end users. These insights also suggest that more nuanced, multidimensional evaluation frameworks are needed to distinguish between perceived and actual privacy risk levels.

### 5.5. Key Data Types Contributing to Privacy Risk

To further explore the drivers of privacy risk, we mapped Control and GDPR questions (shown in Table 3) to specific identity attributes (shown in Table 11) and created composite protection scores for each data type. We then performed both single-variable regression and Lasso feature selection to identify which protections are most predictive of PPA risk. Behavioral data protection consistently emerged as the strongest individual predictor, followed by general PII sharing and data retention/deletion policies.

**Figure 8:**
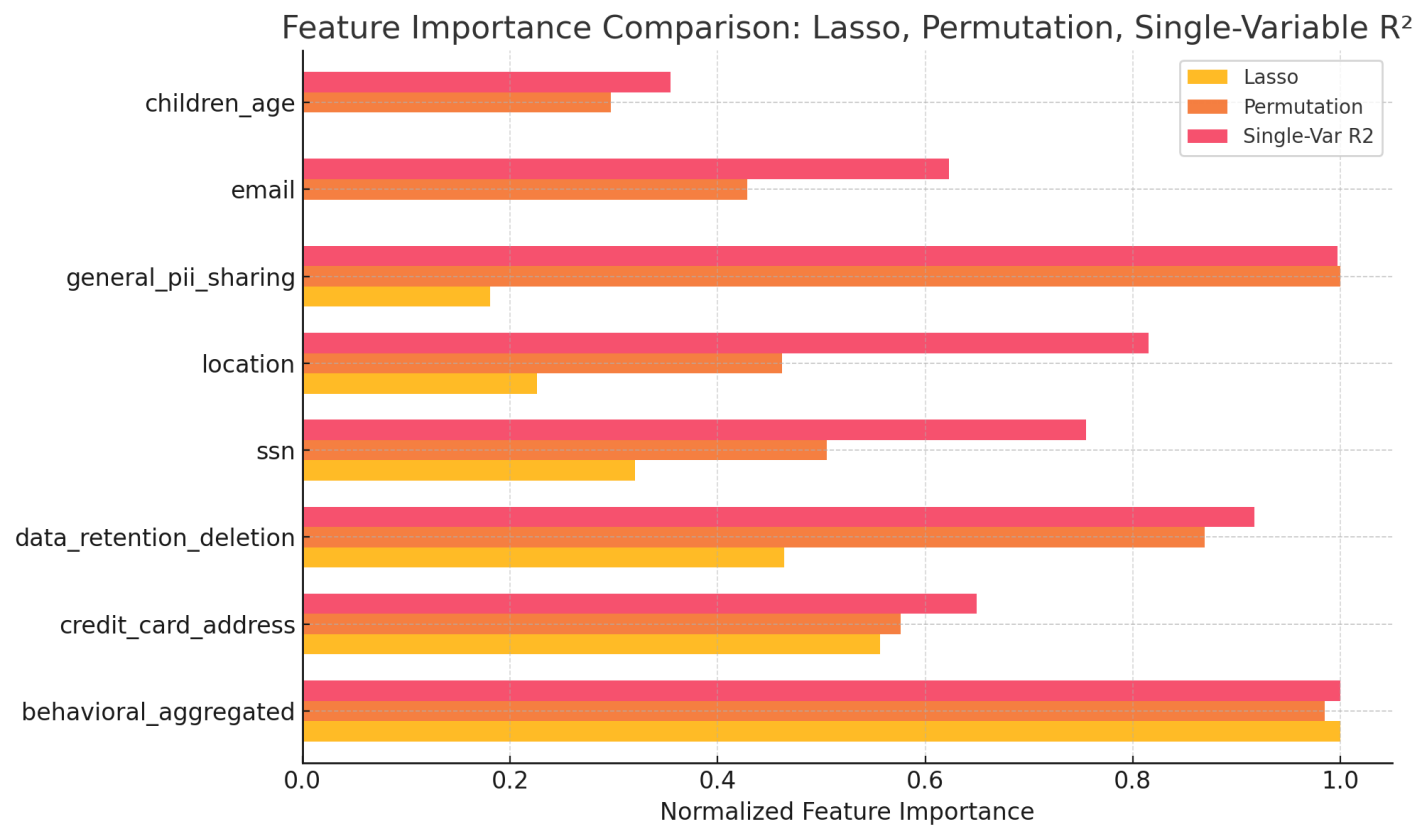
Feature importance comparison for predicting PPA risk using three methods: Lasso regression, permutation importance, and single-variable regression (R^2^). The plot highlights which data type protections have the strongest influence on PPA risk, with higher values indicating greater predictive strength.

To enhance interpretability, we incorporated explainable AI techniques by visualizing not only the feature importance—using Lasso, permutation, and single-variable regression methods—but also the distribution of protection scores for each data type across all apps. As shown in Figure 8, this combined view allows us to see both which data types most strongly influence PPA risk prediction and how well each type of data is protected in practice. This approach provides actionable insights for prioritizing improvements in privacy policies and app practices.

Notably, our analysis pinpoints behavioral data collection and general PII sharing as the top contributors to privacy risk. This suggests that interventions aimed at limiting behavioral tracking and restricting broad data sharing practices could yield the greatest reduction in user risk. Furthermore, the explainable AI feature importance results provide concrete guidance for both app developers and privacy regulators on which policy areas warrant the most attention. By surfacing these specific data types as risk drivers, our work moves beyond general observations to offer actionable recommendations for privacy protection.

### 5.6. App Category Insights

Analysis across application categories revealed that Tools and Productivity apps constituted the largest portions of the sample, as shown by the highest number of apps in these categories. In contrast, Business and Travel apps, although fewer in number, requested the highest average number of permissions per app.

**Figure 9:**
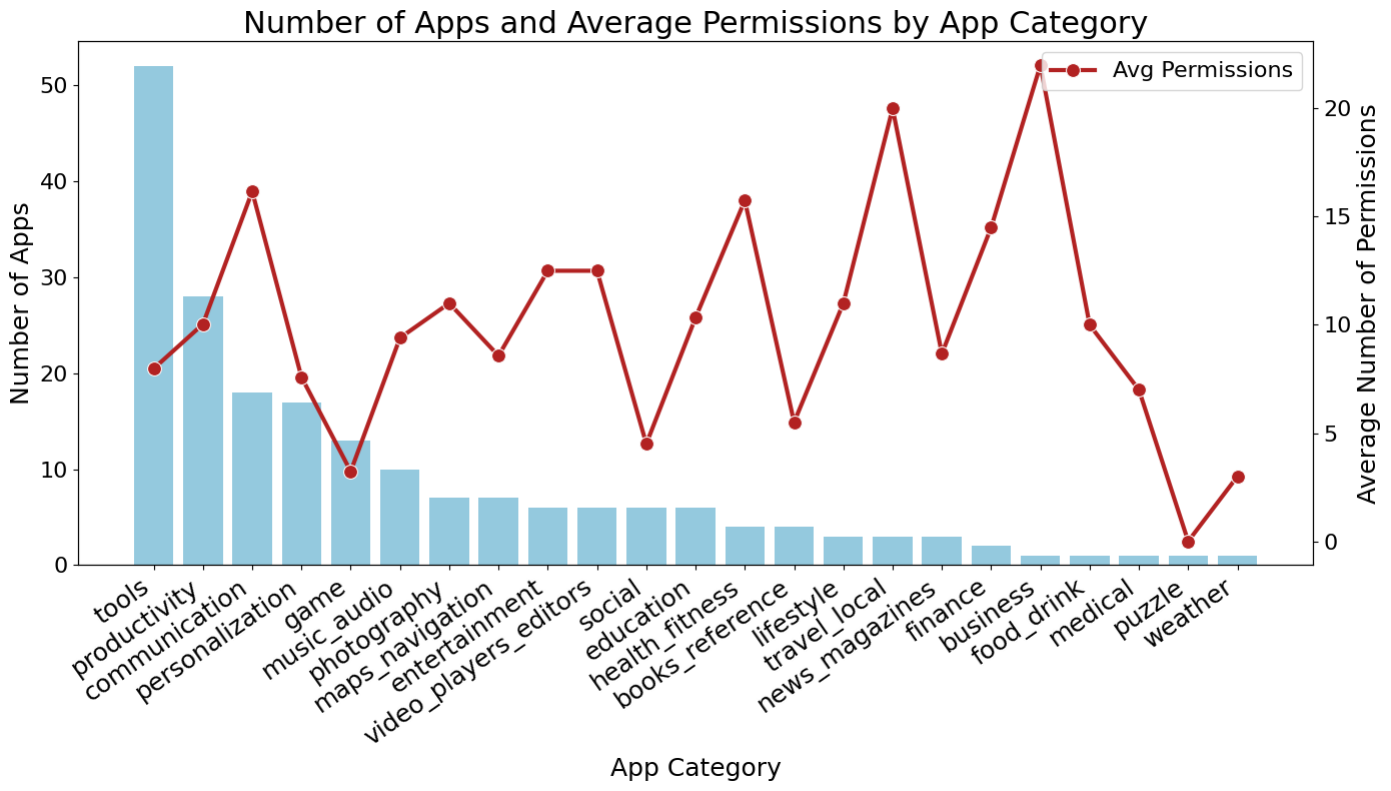
Number of apps and average number of permissions by app category.

Figure 9 shows the distribution of app counts (bars) and average permission usage (line) across categories. These app category insights emphasize that privacy risk is not evenly distributed across the mobile ecosystem. Targeted privacy interventions or regulatory scrutiny may be most effective if focused on categories such as Tools, Communication, productivity, and Business, where both permission requests and high-risk behaviors are concentrated. Recognizing these sectoral differences enables more efficient allocation of privacy resources and more effective user education about category-specific risks.

Overall, our results demonstrate that GDPR and Control compliance scores are insufficient proxies for real-world privacy risks. Behavioral data collection practices, general PII sharing, and data retention mechanisms are primary contributors to elevated risk. Furthermore, app categories vary widely in permission use, with high-risk behaviors concentrated in specific sectors. In summary, our experimental findings demonstrate that technical risk assessments and policy-based scores are often misaligned, with significant implications for both individual privacy protection and broader regulatory strategies. By identifying the most influential data types and app categories, our results offer a roadmap for future privacy risk mitigation in the IoT and mobile app domains.

## 6. Conclusions

This paper presents a practical approach to privacy risk assessment in IoT environments by integrating cycle decomposition into Bayesian networks. We propose a method that addresses the challenges posed by cyclic dependencies in probabilistic models, which are common in real-world privacy risk assessments. Our method enhances the accuracy and efficiency of Bayesian inference by decomposing cyclic structures into acyclic subgraphs, facilitating more reliable privacy risk evaluations.

Through the integration of PrivacyCheck and the UTCID ITAP project, we demonstrate the effectiveness of combining Bayesian networks with entropy-based analysis to assess privacy risks in IoT systems. The inclusion of entropy in our analysis allows for the quantification of uncertainty, providing a deeper understanding of the reliability of privacy risk assessments. By examining the correlation between PPA and PrivacyCheck scores, we highlight the strengths and limitations of existing privacy risk tools and their ability to assess risks associated with various data types.

Our research also contributes to the growing body of work on privacy-preserving technologies by introducing a practical solution for modeling and mitigating identity theft risk in IoT systems. By leveraging Bayesian networks, machine learning, and entropy-based analysis, we offer a framework for privacy risk assessment that can adapt to the complexities of IoT ecosystems.

However, several opportunities for future research remain. Further investigation into the integration of real-time privacy risk assessments and dynamic IoT environments will be essential for improving the applicability of our model in practical settings. Additionally, expanding the scope to incorporate additional privacy-preserving technologies and exploring hybrid approaches that combine Bayesian networks with other machine learning techniques could further enhance the robustness of the risk assessment framework.

In conclusion, this paper provides a scalable method for privacy risk evaluation in IoT systems, contributing to the development of more effective privacy-preserving solutions and fostering greater user control over personal data.

## Figures and Tables

**Table 1:** Comparison of selected privacy risk assessment frameworks and our approach.

Aspect	LINDDUN	CNIL	ISO/IEC 27557	Our Work
Threat Identification	Manual (data flow diagrams, checklists)	Manual (workshops, expert input)	Manual (policy/process review)	Automated (data mining, NLP)
Risk Quantification	Qualitative scoring	Semi-quantitative (risk matrix)	Qualitative	Quantitative (numeric scores)
Automation/ Scalability	Low	Low	Low	High
Attribute-level Analysis	Optional	Possible	No	Yes

**Table 2:** Summary of related work: categories, advantages, and gaps.

Category	Advantages	Gaps / Limitations
Identity Theft Simulation	- Captures complex attacker/defender dynamics - Explores prevention and mitigation strategies - Uses diverse analytical tools (agent-based, ML, statistical)	- Context-specific models - Limited scalability to large or heterogeneous systems
Cyclic Bayesian Network	- Handles cycles in inference - Improves efficiency and tractability - Offers approximate and modular solutions	- May reduce interpretability - Requires specialized knowledge - Some accuracy trade-offs
Privacy Risk Assessment Frameworks	- Systematic threat/risk identification - Widely recognized standards - Support organizational compliance	- Manual, qualitative analysis - Limited automation or scalability - Attribute-level or fine-grained analysis not emphasized
Our Work	- Automated and scalable assessment - Attribute-level, quantitative scoring - Empirical validation with external tools	- Does not replace organizational or contextual risk analysis - May not capture all regulatory or sector-specific nuances

**Table 3:** UTCID PrivacyCheck^™^ questions and scoring method [34].

	User Control	Scores: 100% (Green)	Scores: 50% (Yellow)	Scores: 0% (Red)
1	How well does this website protect your email address?	Not asked for	Used for the intended service	Shared w/third parties
2	How well does this website protect your credit card information and address?	Not asked for	Used for the intended service	Shared w/third parties
3	How well does this website handle your Social Security number?	Not asked for	Used for the intended service	Shared w/third parties
4	Does this website use or share your location?	PII not used for marketing	PII used for marketing	PII shared for marketing
5	Does this website track or share your location?	Not tracked	Used for the intended service	Shared w/third parties
6	Does this website collect PII from children under 13?	Not collected	Not mentioned	Collected
7	Does this website share your information with law enforcement?	PII not recorded	Legal docs required	Legal docs not required
8	Does this website notify or allow you to opt out after changing their privacy policy?	Posted w/opt-out option	Posted w/o opt-out option	Not posted
9	Does this website allow you to edit or delete your information from its record?	Edit/delete	Edit only	No edit/delete
10	Does this website collect or share aggregated data related to your identity or behavior?	Not aggregated	Aggregated w/o PII	Aggregated w/PII
	**GDPR**	**Scores: 100% (Green)**		**Scores: 0% (Red)**
1	Does this website share the user’s information with other websites only upon user consent?	Yes		No/Unanswered
2	Does this website disclose where the company is based/user’s PII will be processed and transferred?	Yes		No/Unanswered
3	Does this website support the right to be forgotten?	Yes		No/Unanswered
4	If they retain PII for legal purposes after the user’s request to be forgotten, will they inform the user?	Yes		No/Unanswered
5	Does this website allow the user the ability to reject their usage of user’s PII?	Yes		No/Unanswered
6	Does this website restrict the use of PII of children under the age of 16?	Yes		No/Unanswered
7	Does this website advise the user that their data are encrypted even while at rest?	Yes		No/Unanswered
8	Does this website ask for the user’s informed consent to perform data processing?	Yes		No/Unanswered
9	Does this website implement all of the principles of data protection by design and by default?	Yes		No/Unanswered
10	Does this website notify the user of security breaches without undo delay?	Yes		No/Unanswered

**Table 4:** Summary of symbols and notation used throughout the methodology.

Symbol	Description
*i*	Identity attribute
P(i)	Prior probability of exposure of attribute *i*
L(i)	Liability value (property loss) associated with *i*
A(i)	Set of ancestor attributes of *i* in the BN
D(i)	Set of descendant attributes of *i* in the BN
$P(i\;|\;i_{k})$	Probability of *i* given ancestor $i_{k}$ is exposed
α(i)	Accessibility of *i* (Equation (5))
β(i)	Post Effect of *i* (Equation (6))
E(i)	Expected loss for *i* (Equation (7))
S(i)	Normalized risk score for *i*
p	Probability distribution vector for *X* (i.e., $p_{i}=P\left(X=x_{i}\right)$)
H(X)	Shannon entropy of random variable *X*
H(p)	Shannon entropy of probability distribution p
$X_{q}$	Random variable for PrivacyCheck question *q*
$H_{\mathrm{tot}}$	Sum of entropies across all PrivacyCheck questions

**Table 5:** Popular open-source apps.

App	PPA Scores (%)	ImmuniWeb
Wiki	43.63	Low
Firefox Focus	47.99	Low
Kodi	48.79	Low
QsmFurthermore,	54.51	Low
Duckduckgo	67.39	Medium
OpenVPN	68.92	Medium
Signal Private Messenger	69.32	Medium
Ted	71.82	Low
Blockchain Wallet	73.67	Medium
Telegram	73.99	Medium

**Table 6:** Five-fold cross-validation accuracy for each privacy factor.

Privacy Factor	Accuracy (%)
Contact Information Sharing	73
PII Usage	72
Third-Party Sharing	63
Data Retention	60
Location Data	60
Data Breach Notification	57
PII Collection Purpose	57
Children’s Privacy	52
Policy Change Notification	44
Data Aggregation/Profiling	40

**Table 7:** PrivacyCheck coverage and accuracy compared with other privacy policy analysis tools.

Tool	Coverage (Out of 50)	Average Accuracy (%)
PrivacyCheck	50	60
Privee	50	74
ToS;DR	14	57
Usable Privacy	14	59
P3P	1	100

**Table 8:** Comparison of CNIL privacy risk assessment steps and corresponding elements in PPA and PrivacyCheck.

CNIL Step	PPA Score Approach	PrivacyCheck Score Approach
Identify Threats	Models the exposure of identity attributes as distinct privacy threats for each app or device.	Assesses the presence and coverage of privacy policy controls related to potential threats, such as unauthorized sharing or processing.
Assess Severity of Impact	Assigns a loss value to each identity attribute, reflecting its sensitivity and potential consequences of exposure.	Addresses severity indirectly, as some policy questions focus on sensitive data types (e.g., SSN, location).
Estimate Likelihood of Occurrence	Calculates the probability of exposure for each attribute, based on observed or modeled data flows in the ecosystem.	Higher PrivacyCheck scores indicate stronger policy controls, which are associated with a lower likelihood of privacy risks occurring.
Combine to Assess Risk	Aggregates probability and loss values into an overall risk score for each app (e.g., risk = probability × loss).	Provides a composite policy score as a proxy for the app’s overall risk mitigation practices and compliance.

**Table 9:** Statistics for each subgraph obtained through cycle decomposition. PC stands for prior change. EW stands for edge weight. AC stands for accessibility. PE stands for Post Effect. PR stands for privacy risk.

Subgraph	PC	EW	AC	PE	PR
S1	0.031	0.915	0.052	0.216	0.896
S2	0.169	1.00	0.487	0.019	0.901
S3	0.806	0.999	0.333	0.434	0.934
S4	0.019	0.914	0.445	0.107	0.913
S5	0.007	0.700	0.405	0.146	0.917
S6	0.524	0.897	0.336	0.035	0.899
S7	0.058	0.800	0.515	0.379	0.903
S8	1.00	0.901	0.228	0.172	0.914
S9	0.089	0.885	0.269	0.320	0.900
S10	0.552	0.781	0.336	0.714	1.00
S11	0.086	0.786	0.430	0.001	0.898
S12	0.069	0.699	1.00	0.148	0.922
S13	0.012	0.584	0.268	0.513	0.973
S14	0.001	0.896	0.353	0.055	0.902
S15	0.126	0.900	0.568	1.00	0.968
AVG	0.237	0.844	0.402	0.284	0.923

**Table 10:** Multiple regression analysis predicting PPA risk from PrivacyCheck scores.

Feature	Coefficient	*p*-Value
Control Score	+0.123	0.267
GDPR Score	+0.273	<0.001

**Table 11:** Mapping of data types to relevant PrivacyCheck questions.

Data Type	Relevant Questions
Email	Control Q1, GDPR Q1
Credit Card/Address	Control Q2, GDPR Q7
SSN	Control Q3, GDPR Q5
Location	Control Q5, GDPR Q1, GDPR Q7
Children’s Data/Age	Control Q6, GDPR Q6
General PII Sharing	Control Q4, Control Q7, GDPR Q2, GDPR Q8
Data Retention / Deletion	Control Q9, GDPR Q3, GDPR Q4
Behavioral/Aggregated	Control Q10, GDPR Q9

## Data Availability

The datasets presented in this article are not readily available because the data are part of an ongoing study. Requests to access the datasets should be directed to the corresponding author(s).

## Abbreviations

The following abbreviations are used in this manuscript:

UTCID	University of Texas at Austin, Center for Identity
ITAP	The Identity Threat Assessment and Prediction
PII	Personally Identifiable Information
PPA	Personal Privacy Assistant
IoT	Internet of Things
NLP	Natural Language Processing
